# FGF-2 enhances fibrogenetic changes in TGF-β2 treated human conjunctival fibroblasts

**DOI:** 10.1038/s41598-022-20036-7

**Published:** 2022-09-26

**Authors:** Yuri Tsugeno, Masato Furuhashi, Tatsuya Sato, Megumi Watanabe, Araya Umetsu, Soma Suzuki, Yosuke Ida, Fumihito Hikage, Hiroshi Ohguro

**Affiliations:** 1 Departments of Ophthalmology, Sapporo Medical University School of Medicine, Sapporo, Japan; 2 Departments of Cardiovascular, Renal and Metabolic Medicine, Sapporo Medical University School of Medicine, Sapporo, Japan; 3 Departments of Cellular Physiology and Signal Transduction, Sapporo Medical University School of Medicine, Sapporo, Japan

**Keywords:** Cell biology, Medical research

## Abstract

The objective of the current study was to examine the effects of fibroblast growth factor-2 (FGF-2) on conjunctival fibrogenesis that was induced by the presence of transforming growth factor-β2 (TGF-β2). Two-dimension (2D) and three-dimension (3D) cultured human conjunctival fibroblasts (HconF) were used for this purpose. The 2D and 3D cultured HconF were characterized by transendothelial electrical resistance (TEER) and FITC dextran permeability measurements (2D), real-time metabolic analyses (2D), size and stiffness measurements (3D), and the mRNA expression of extracellular matrix molecules, their modulators, Tissue inhibitor of metalloproteinases and matrix metalloproteinases and ER-stress related genes (2D and 3D). FGF-2 significantly increased planar proliferation, as evidenced by TEER values and FITC dextran permeability, and shifted glucose metabolism to the energetic phenotype of 2D HconF cells, and the stiffness of the 3D spheroids, and these effects were further enhanced in the presence of TGF-β2. Analyses of the expression of possible candidate molecules involved in cell architecture and stress indicated that some additive effects caused by both factors were also recognized in some of these molecules. The findings reported herein indicate that the FGF-2, either along or additively with TGF- β2 increased the fibrogenetic changes on the plane as well as in the spatial space of HconF cells.

## Introduction

The environment of the human ocular surface is maintained in their healthy condition by conjunctiva, a biological barrier. This condition, however, is sometimes affected by several ocular surface diseases in addition to surgical intervention, which are associated with subconjunctival fibrogenetic changes^1^. Therefore, to obtain optimal surgical outcomes after the occurrence of several ocular surface-related diseases, including glaucoma, such subconjunctival fibrosis needs to be properly regulated^2–8^. Concerning the mechanisms responsible for both the normal wound-healing as well as pathologic fibrosis, fibroblasts and several factors, including cytokines and growth factors are largely involved^9,10^. Among some of these related factors, it is known that transforming growth factor-beta (TGF-β) functions to regulate nearly all wound healing related mechanisms^10^, and, in fact, TGF-β has been shown to facilitate the trans-differentiation of fibroblasts into myofibroblasts^9,11,12^. It has also known that myofibroblasts are removed from the wound area by apoptosis during the normal wound healing process. However, in contrast, if this normal wound repairing process is perturbed, pathological fibrosis caused by myofibroblasts develops, thus leading to additional scar formation^11,13^. Thus, a strategy for preventing this conversion of fibroblasts into myofibroblasts is pivotal for maintaining healthy planar proliferations of human conjunctiva^7,14,15^.

Fibroblast growth factor-2 (FGF-2), a growth factor that is involved in wound repair, is a 146-amino acid polypeptide molecule that initiates the production of mesoderm- and ectoderm-derived cells, including fibroblasts, endothelial cells, and epithelial cells^16–18^. It has been shown that the application of growth factors enhances wound repair in skin^19^. In addition, FGF-2 has also been used to repair damage to brain neurons^20^, corneal injuries^21^, and facial nerve injuries^22^ and to promote scarless healing^23^. However, the FGF-2-induced effects on TGF-β2-related conjunctival fibrogenetic changes remain controversial. In this area, Kay et al. reported that FGF-2 stimulates TGF-β-mediated cell proliferation in rabbit subconjunctival fibroblasts^24^. On the contrary, Matsumura et al. reported that FGF-2 induces the of the TGF-β-mediated trans-differentiation within human conjunctival fibroblast (HconF) cells^25^.

In the present study, to clarify this ambiguity, we examined the FGF induced effects on TGF-β2-untreated or -treated two-dimensional (2D) and three-dimensional (3D) HconF cell cultures, which have previously been shown to be suitable in vitro conjunctival models for fibrogenetic changes on the plane or in the spatial space, respectively^26^.

## Methods

### 2D and 3D cultures of human conjunctival fibroblasts (HconF)

Commercially available HconF cells (ScienCell Reserch laboratories, CA USA) were cultured, then maintained in 150 mm 2D culture dishes and subjected to a series of analyses including transendothelial electron resistance (TEER), fluorescein isothiocyanate (FITC)-dextran permeability measurements and the measurement of real-time cellular metabolic functions as described below. Alternatively, 2D cultured HconF cells were also further processed to prepare 3D HconF spheroids during a 6-day culture period as described in previous reports^26–29^. For evaluating drug induced effects, 5 ng/mL solutions of TGF-β2, 10 ng/ml solutions of FGF-2, or both was added to 2D or 3D cultured Hcon F cells at Days 1 through 6. The dosages of the TGF-β2 and FGF-2 used in the present study were based on data reported in a previous study^25,26^.

### Transendothelial electron resistance (TEER) and FITC-dextran permeability measurements of 2D cultured HTM cells

The TEER values for HconF cell monolayers were determined using a TEER plate (0.4 μm pore size and 12 mm diameter; Corning Transwell, Sigma-Aldrich) and an electrical resistance system (KANTO CHEMICAL CO. INC., Tokyo, Japan) as described in a previous study^26,30^. Alternatively, FITC-dextran permeability measurements were conducted by measuring the fluorescence intensity of the amount of FITC that permeated through the membrane from the basal compartment to the apical compartment during a period of 60 min, as described in a recent report^31^.

### Measurement of real-time cellular metabolic functions

The rates of oxygen consumption rate (OCR) and extracellular acidification (ECAR) of 2D HconF cells were measured using Seahorse XFe96 Bioanalyzer (Agilent Technologies) as described previously with minor modifications^32,33^. Briefly, 20 × 10^3^ 2D HconF cells were placed in wells of a 96-well assay plate as follows; (1) non-treated control (NT), (2) treated with TGF-β2, (3) treated with FGF-2 and (4) treated with TGF-β2 and FGF-2. After replacing the culture medium with Seahorse XF DMEM assay medium (pH 7.4, Agilent Technologies, #103,575–100) supplemented with 5.5 mM glucose, 2.0 mM glutamine, and 1.0 mM sodium pyruvate, basal OCR and ECAR values were determined using a Seahorse XFe96 Bioanalyzer and thereafter, the samples were further analyzed after supplementation with 2.0 μM oligomycin, 5.0 μM carbonyl cyanide p-trifluoromethoxyphenylhydrazone (FCCP), 1.0 μM rotenone and antimycin A, and 10 mM 2-deoxyglucose (2-DG). The OCR and ECAR values were normalized to the amount of protein per well.

### Measurement of the size and solidity of 3D HconF spheroids

For evaluating physical properties, the mean size and stiffness, of the 3D ConF spheroids were determined by measuring their largest cross-sectional area (CSA) using an inverted microscope (Nikon ECLIPSE TS2; Tokyo, Japan), a micro-squeezer (MicroSquisher, CellScale, Waterloo, ON, Canada) as reported in a previous study^27,29^.

### Immunocytochemistry of 2D and 3D cultured HconF cells

The immunocytochemistry of the 2D and 3D cultured HconF cells was evaluated using 1^st^ antibodies; an anti-human COL1, COL4, COL6, FN or αSMA rabbit antibody (1:200 dilutions), a goat anti-rabbit IgG (488 nm, 1:1000 dilutions), phalloidin (594 nm, 1:1000 dilutions) and DAPI (1:1000 dilutions), and confocal immunofluorescent images were obtained, as described in a recent report^28,34^.

### Other analytical Methods

Total RNA was extracted from the 2D or 3D cultured HconF cells and reverse transcription and real-time PCR were carried out as previously reported^28,34^ using specific primers and probes (supplemental Table 1).

All statistical analyses were performed using Graph Pad Prism 8 (GraphPad Software, San Diego, CA) as described in a recent report^28,34^.

## Results

### Effects of FGF-2 toward the fibrogenic properties of the2D cultured HconF cells

**Figure 1:**
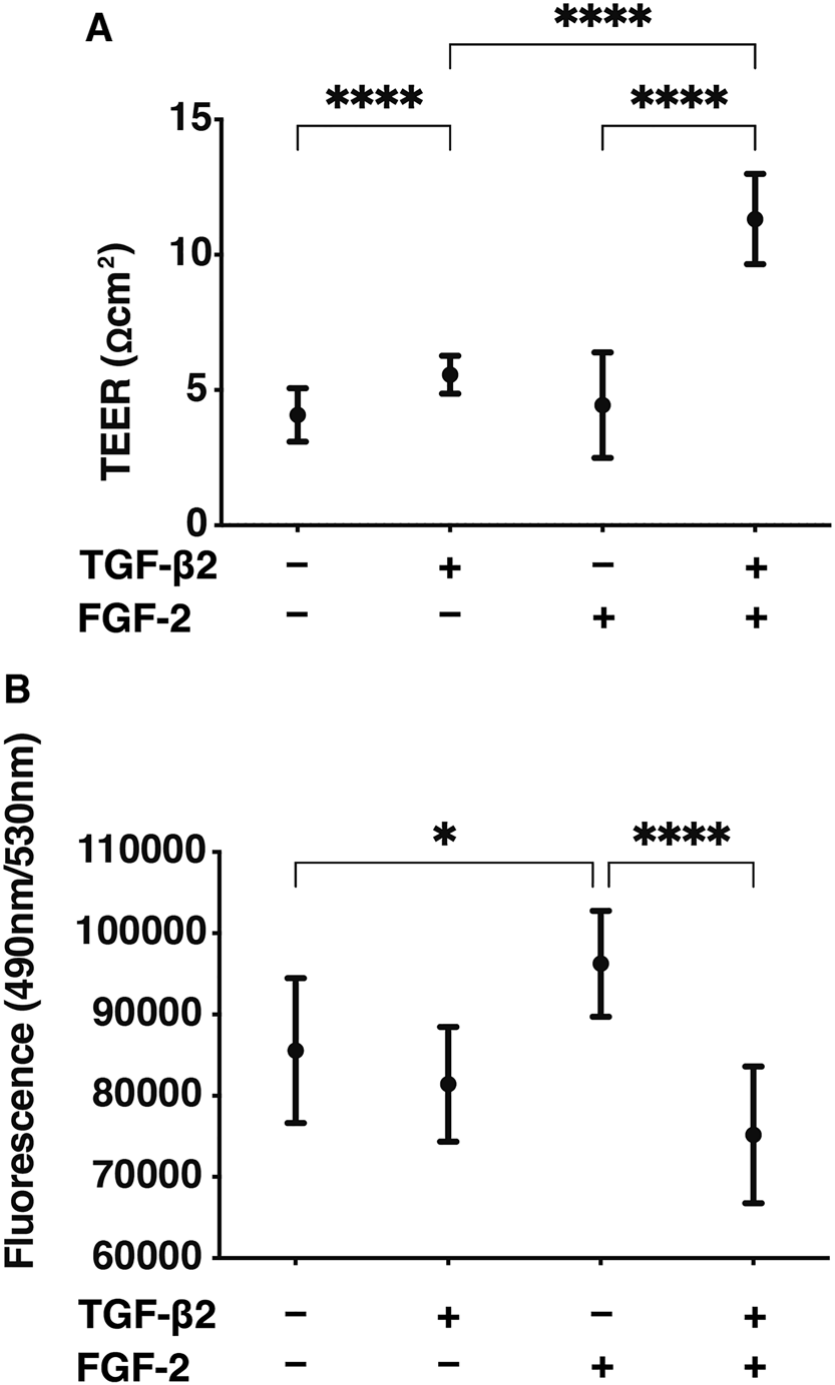
Effects of FGF-2 on measurements by transendothelial electrical resistance (TEER) and FITC-dextran permeability of 2D HconF monolayers in the absence and presence of TGF-β2. The 2D HconF cell monolayer was treated with a 5 ng/ml solution of TGF-β2 in the absence or presence of 10 ng/ml FGF-2 with an untreated sample as the control. The 2D cultures of HconF monolayers at Day 6 were subjected to planar proliferation analyses by electric resistance (Ω cm^2^) measurements using TEER (panel A) and FITC-dextran permeability (panel B). All experiments were performed in triplicate using fresh preparations. Data are presented as the arithmetic mean ± the standard error of the mean (SEM). *P < 0.05, **P < 0.01, ****P < 0.001 (ANOVA followed by a Tukey’s multiple comparison test).

**Figure 2:**
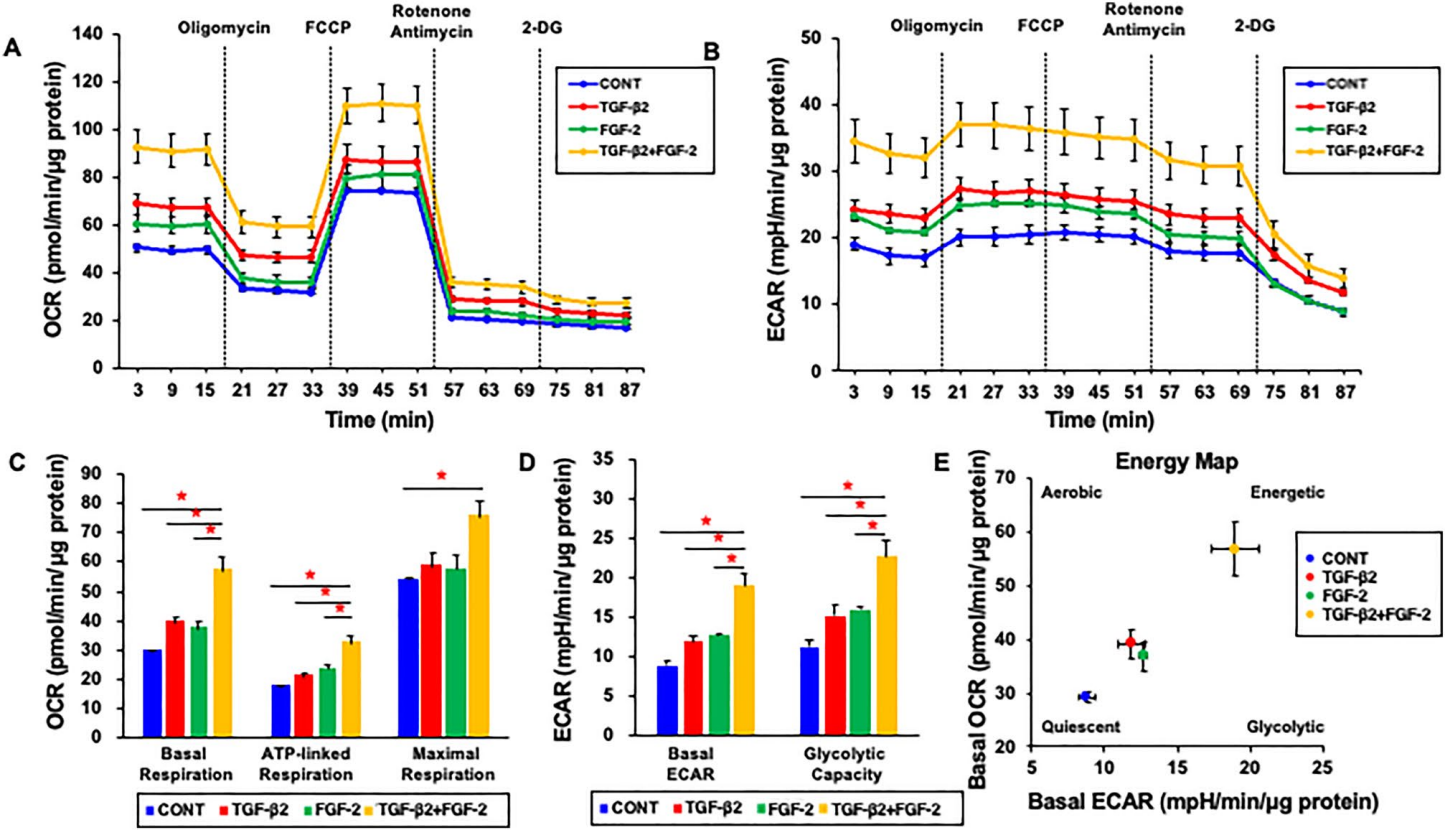
Effects of FGF-2 on the cellular metabolic phenotype of the 2D HconF cells in the absence or presence of TGF-β2. 2D HconF cells were treated with a 5 ng/ml solution of TGF-β2 in the absence or presence of 10 ng/ml FGF-2 with an untreated sample as the control. At Day 6, the samples were subjected to a real-time metabolic function analysis using a Seahorse XFe96 Bioanalyzer. (**A**, **B**) Basal OCR and ECAR were measured, and thereafter they were further measured by subsequent supplementation with oligomycin (complex V inhibitor), FCCP (a protonphore), and rotenone/antimycin A (complex I/III inhibitors) and 2DG (hexokinase inhibitor). (**C**, **D**) Basal respiration is calculated by subtracting OCR with rotenone/antimycin A from OCR at baseline. ATP-linked respiration was defined by the difference in OCR after the addition of oligomycin. Maximal respiration was calculated by subtracting OCR with rotenone/antimycin A from OCR with FCCP. Basal ECAR and Glycolytic capacity were defined by subtracting ECAR with 2-DG from ECAR at baseline and ECAR with oligomycin, respectively. (**E**) Energy map for cells treated or not treated with TGF-β2 in the absence or presence of FGF-2. All experiments were performed in triplicated using fresh preparations. Data are presented as the mean ± the standard error of the mean (SEM). *P < 0.05 (ANOVA followed by a Tukey’s multiple comparison test).

In our previous study, we reported on the development of a suitable in vitro model that replicates conjunctival fibrogenesis using TGF-β2 treated 2D and 3D cultured HconF cells, which mimic the TGF-β2 related myofibroblast-induced changes on the surface plane as well as in the subepithelial spatial space, respectively^26^. In the present study, we examined the effects of FGF-2, a possible anti-fibrotic factor^18^, on these TGF-β2 treated 2D and 3D cultured HconF models. In prior to the current study, positive expressions of FGR-2 receptor (FGFR) within our HconF preparations were confirmed (Supplemental Fig. 1). To study the effects of FGF-2 on the TGF-β2-induced changes on the surface of myofibroblasts, the planar proliferation of 2D HconF monolayers were evaluated by TEER measurement and FITC-dextran permeability (Fig. 1). A significant increase in the TEER values and a relative decrease in FITC-dextran permeability were observed upon exposure to TGF-β2. While, in contrast, FGF-2 induced a slight increase in FITC-dextran permeability. However, and quite interestingly, the presence of both factors resulted in a substantial increase in the TEER values and a decrease in FITC-dextran permeability. Such additive effects by both factors were also observed in the real-time cellular metabolic analysis of 2D HconF cells. That is, although both the oxygen consumption rate (OCR), which reflects mitochondrial function, and the extracellular acidification rate (ECAR), which reflects glycolytic function, were not affected by each factor alone, but these indices were significantly elevated when both factors were present (Fig. 2). These results rationally suggest that the activated biological activities by the presence of both factors related to the increase in planar proliferation may require the metabolic state to be converted to a more energetic phenotype.

### Effects of FGF-2 toward the fibrogenic properties of the 3D HconF spheroids

**Figure 3:**
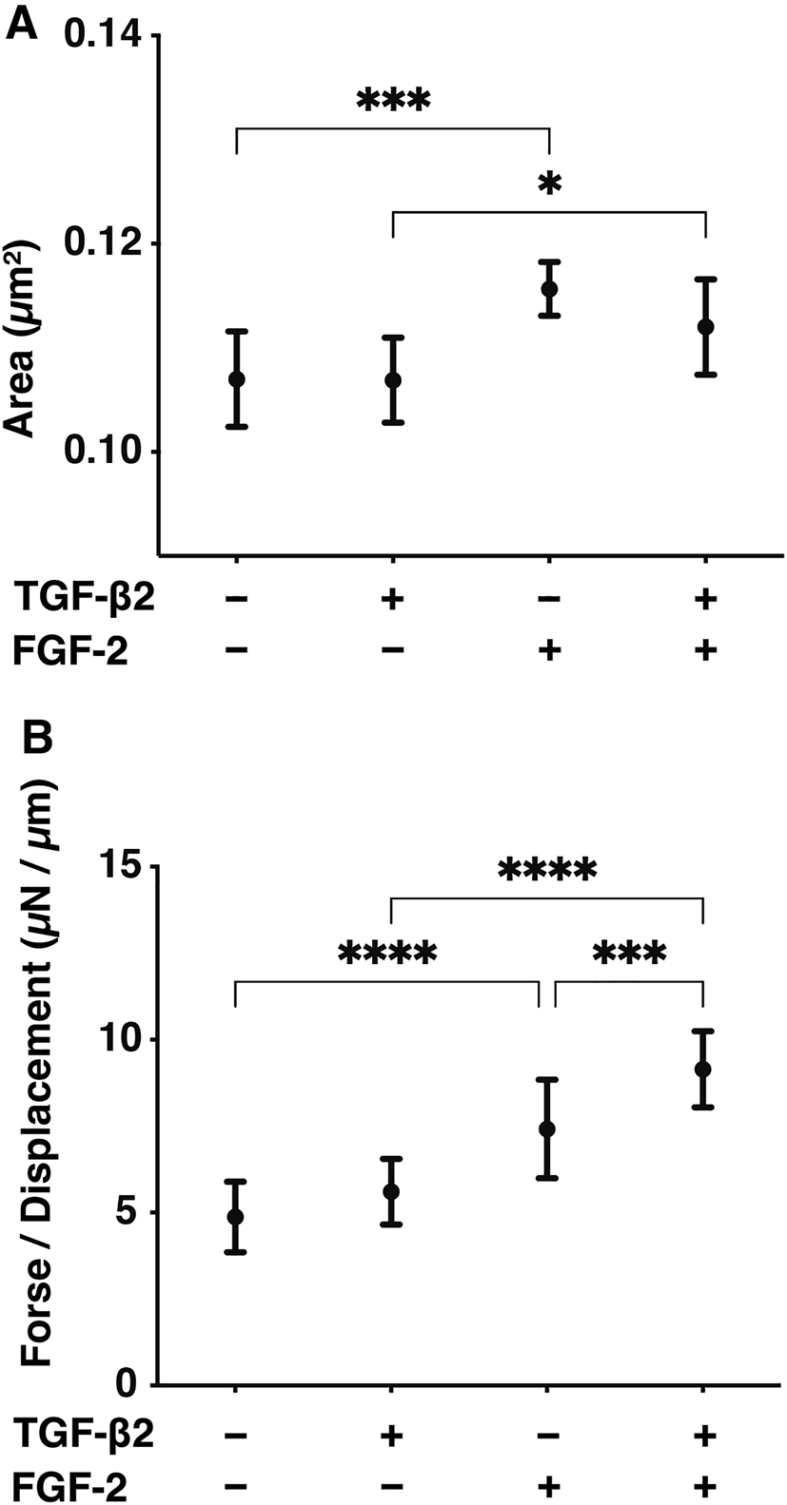
Effects of FGF-2 on the physical properties, sizes and stiffness of 3D HconF spheroids in the absence and presence of TGF-β2. 3D HconF spheroids at Day 6 were treated with a 5 ng/ml solution of TGF-β2 in the absence or presence of 10 ng/ml FGF-2 with an untreated sample as the control. Mean sizes of 3D HconF spheroids were measured and the plotted data are shown in panel A. The physical solidity of their 3D HconF spheroids was analyzed by a micro-squeezer (panel A, scale bar: 100 µm), and the force required to produce a 50% deformity of a single spheroid during a period of 20 s was plotted in panel B. All experiments were performed in triplicate using fresh preparations consisting of 16 spheroids each. Data are presented as the arithmetic mean ± standard error of the mean (SEM). **P < 0.01, ****P < 0.001 (ANOVA followed by a Tukey’s multiple comparison test).

To study the FGF-2 induced effects on the TGF-β2 related myofibroblast-induced changes in the subepithelial spatial space, the physical properties, size and stiffness of the 3D HconF spheroids were evaluated. As shown in Fig. 3, the mean sizes of the 3D HconF spheroids were significantly increased by FGF-2 in both the presence and absence of TGF-β2 (Fig. 3A). However, in contrast, the physical stiffness of the 3D HconF spheroids was also increased by the presence of FGF-2 and these effects were further enhanced by the presence of TGF-β2, as observed in the 2D HconF cells as above (Fig. 3B). These collective findings indicate that FGF-2 significantly facilitates the TGF-β2-related myofibroblast-induced changes on the surface plane as well as in the subepithelial spatial space, as was suggested by Kay et al.^24^.

### Effects of FGF-2 toward the expressions of several ECM proteins, their modulators and ER stress related factors of the 2D and 3D cultured HconF cells

**Figure 4:**
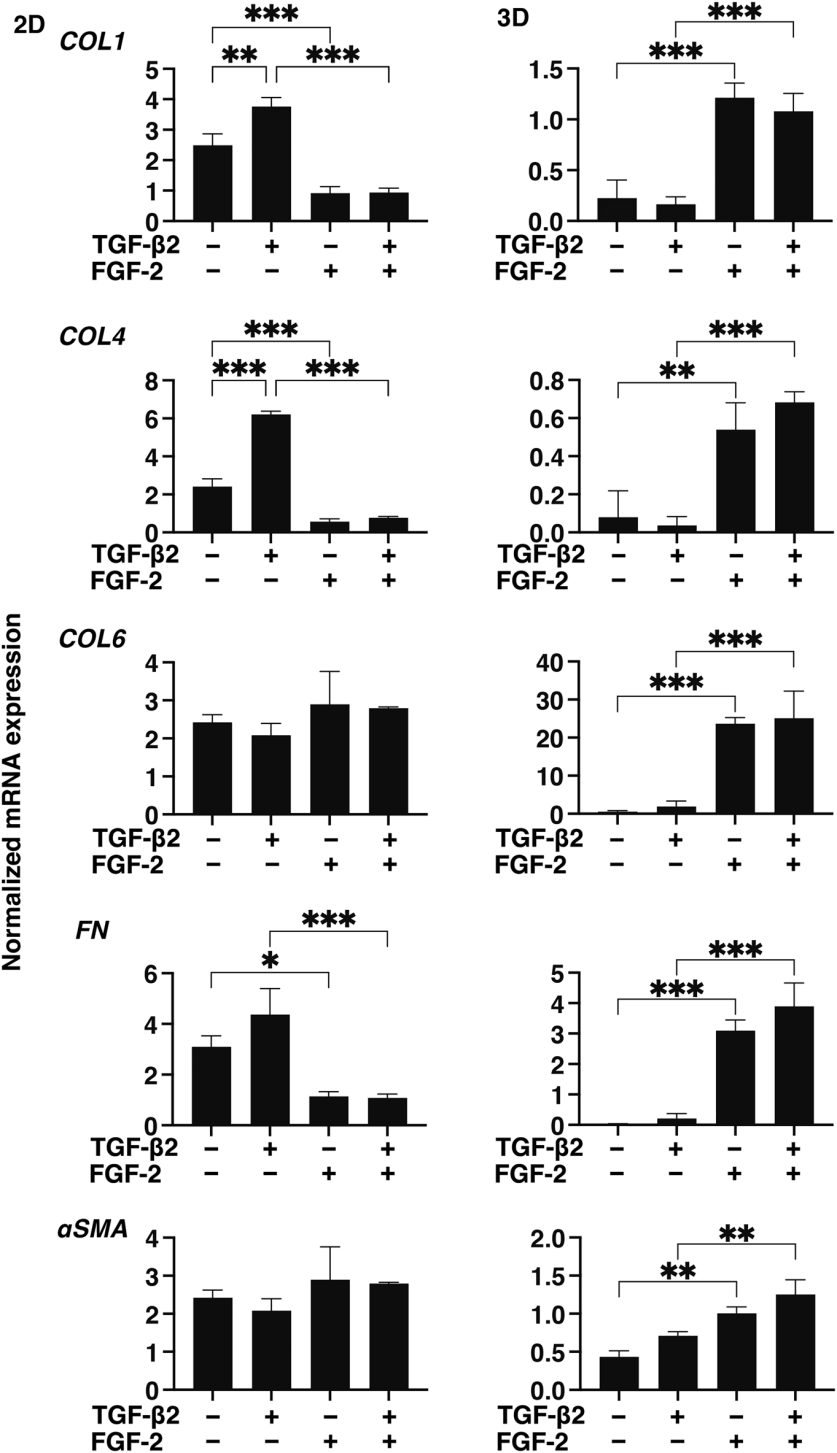
Effects of FGF-2 on mRNA expression of ECMs of 2D and 3D cultured HconF cells in the absence or presence of TGF-β2. 2D and 3D HconF cells were treated with a 5 ng/ml solution of TGF-β2 in the absence or presence of 10 ng/ml FGF-2 with an untreated sample as the control, and at Day 6 each sample was subjected to qPCR analysis and the expression of mRNA in ECMs, *COL1*, *COL4*, *COL6*, *FN,* and *aSMA* were estimated. All experiments were performed in duplicate using 3 different confluent 6-well dishes (2D) or 15 freshly prepared 3D HconF spheroids (3D) in each experimental condition. Data are presented as the arithmetic mean ± the standard error of the mean (SEM). *P < 0.05, **P < 0.01, ***P < 0.005 (ANOVA followed by a Tukey’s multiple comparison test).

**Figure 5:**
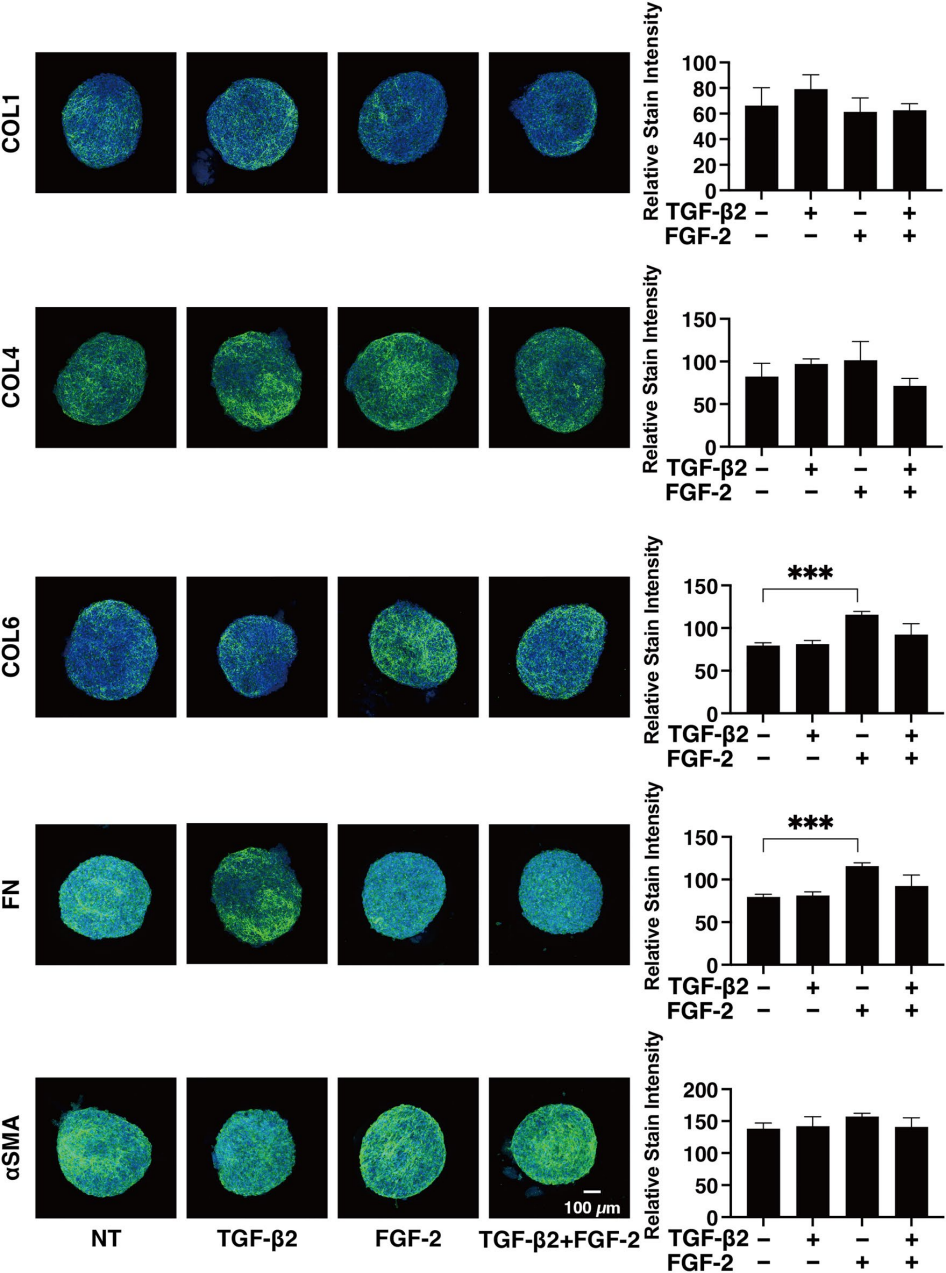
Immunolabeling of ECMs of the 3D HconF sphenoids. 3D HconF spheroids were treated with a 5 ng/ml solution of TGF-β2 in the absence or presence of 10 ng/ml FGF-2 with an untreated sample as the control, and at Day 6, each sample was subjected to immunostaining for *COL 1*, *COL 4*, *COL 6*, *FN* and *a-SMA*. All experiments were performed in duplicate using fresh preparations (*n* = 5). Representative images are shown in left panels and relative staining intensities were plotted in right panels. Data are presented as the arithmetic mean ± standard error of the mean (SEM). **P < 0.01, ***P < 0.005 (ANOVA followed by a Tukey’s multiple comparison test).

**Figure 6:**
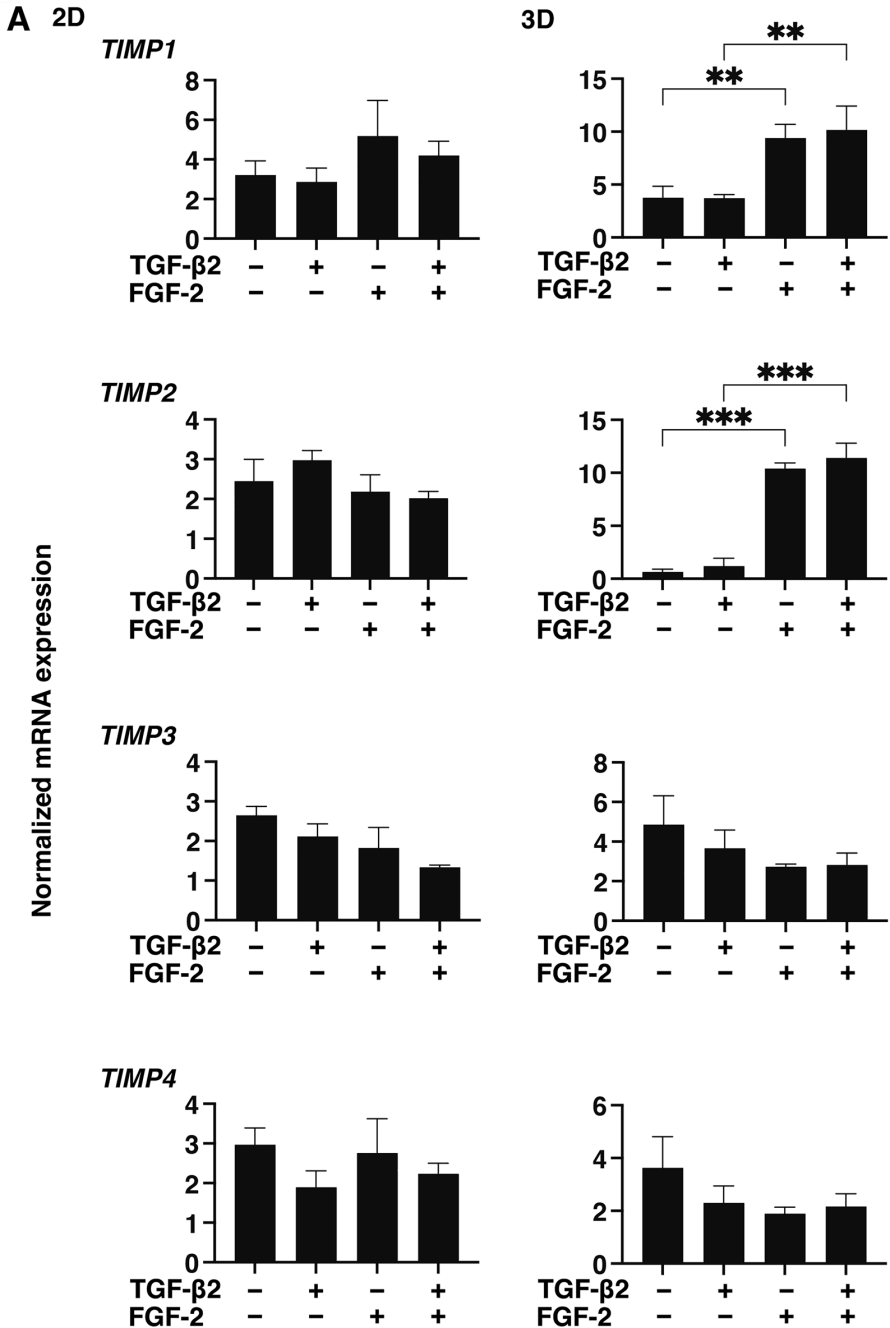

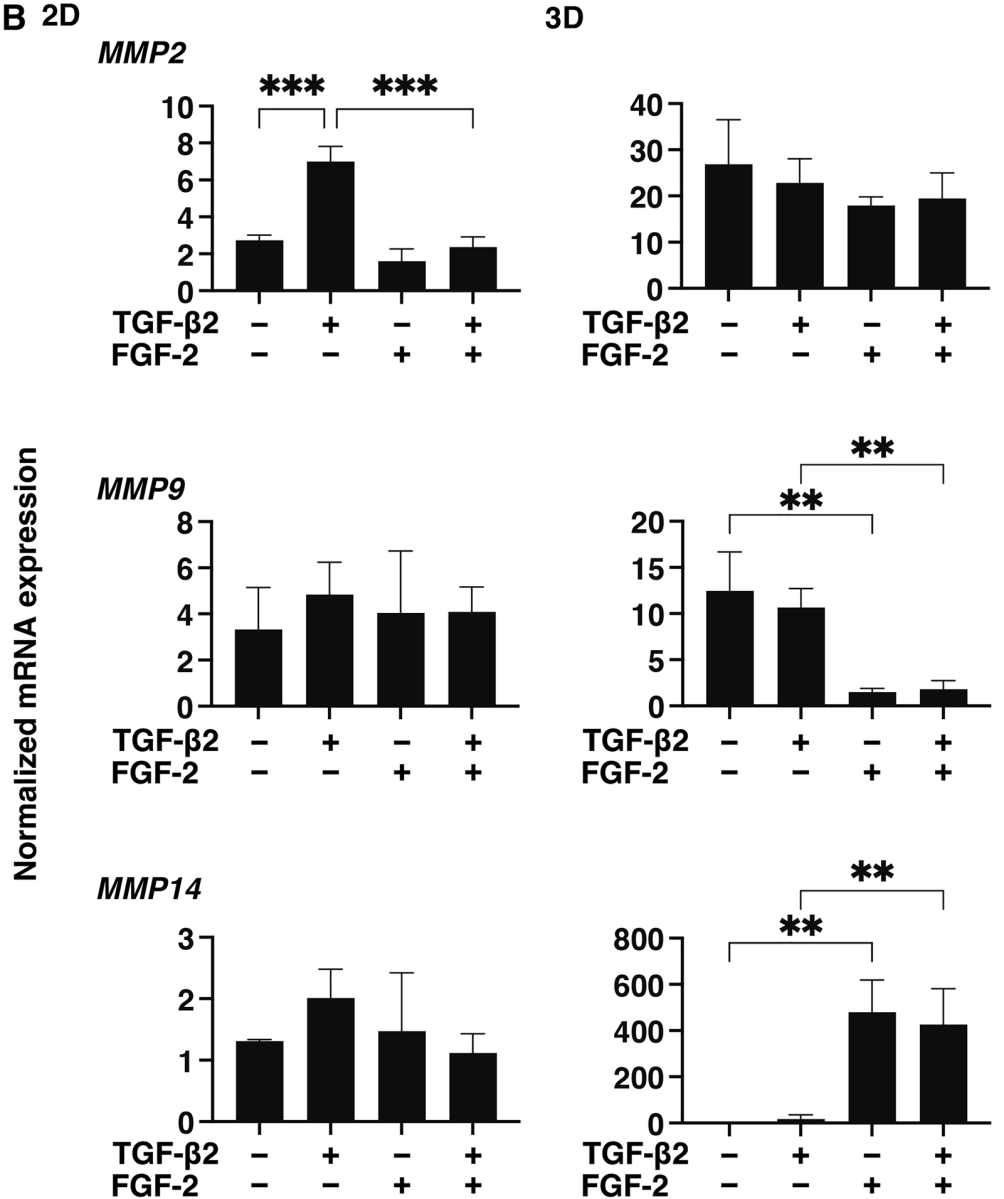
Effects of FGF-2 on the mRNA expression of TIMPs and MMPs of 2D and 3D cultured HconF cells in the absence and presence of TGF-β2. 2D and 3D cultured HconF cells were treated with a 5 ng/ml solution of TGF-β2 in the absence or presence of 10 ng/ml FGF-2 with an untreated sample as the control. Each 2D cell sample and 3D HconF spheroids at Day 6 was subjected to qPCR analysis to estimate the expression of mRNA in *TIMP1-4* (**A**) and *MMP2, 9* and *14* (**B**). All experiments were performed in duplicate using 3 different confluent 6-well dishes (2D) or 15 freshly prepared 3D HconF spheroids (3D) in each experimental condition. Data are presented as the arithmetic mean ± the standard error of the mean (SEM). *P < 0.05, **P < 0.01 (ANOVA followed by a Tukey’s multiple comparison test).

**Figure 7:**
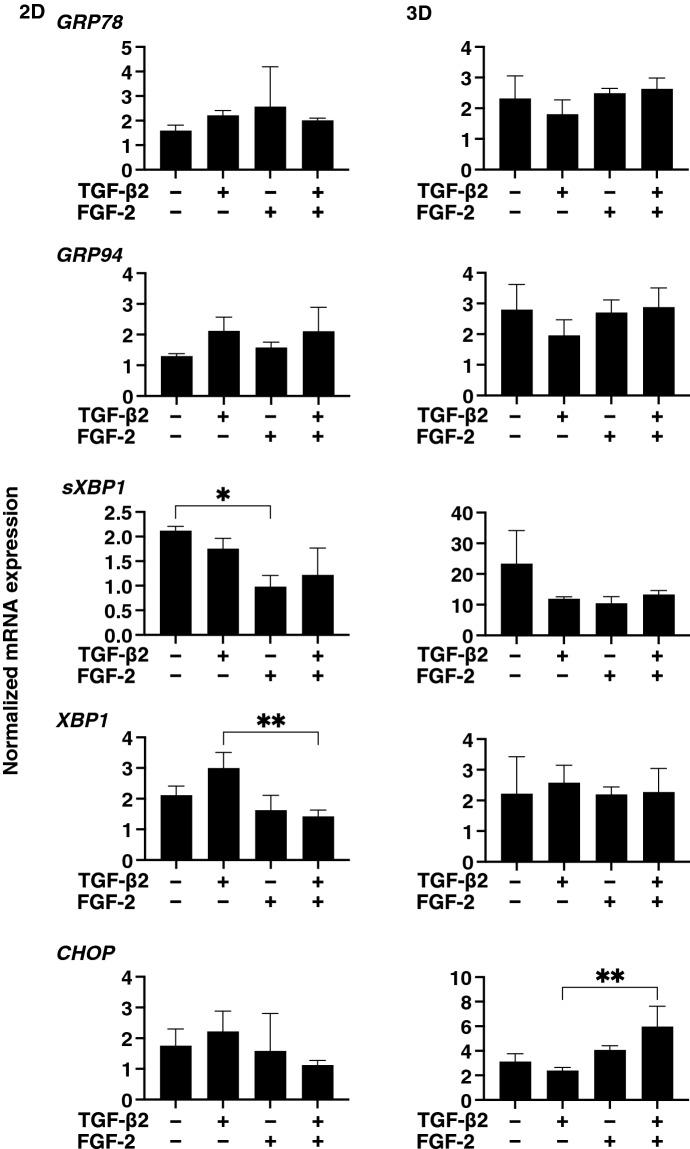
Effects of FGF-2 on mRNA expression of ER-stress related factors of 2D and 3D cultured HconF cells in the absence and presence of TGF-β2. 2D and 3D cultured HconF cells were treated with a 5 ng/ml solution of TGF-β2 in the absence or presence of 10 ng/ml FGF-2 with an untreated sample as the control. Each 2D cell sample and 3D HconF spheroids at Day 6 was subjected to qPCR analysis to estimate the expression of ER stress related genes including the glucose regulator protein (GRP)78, GRP94, the X-box binding protein-1 (XBP1), spliced XBP1 (sXBP1) and CCAAT/enhancer-binding protein homologous protein (CHOP). All experiments were performed in duplicate using 3 different confluent 6-well dishes (2D) or 15 freshly prepared 3D HconF spheroids (3D) in each experimental condition. Data are presented as the arithmetic mean ± the standard error of the mean (SEM). *P < 0.05, **P < 0.01 (ANOVA followed by a Tukey’s multiple comparison test).

To examine this issue in more detail and to elucidate the currently unknown mechanisms responsible for causing this FGF-2 induced enhancement in the TGF- β2-related myofibroblast induced changes in 2D and 3D cultured HconF cells, the expression of some major ECM proteins including collagen1 (COL1), COL4, COL6, fibronectin (FN) and α smooth muscle actin (αSMA) were determined by qPCR analysis and immunocytochemistry. As shown in the qPCR analysis (Fig. 4), there were differences in the TGF- β2 induced up-regulation between the 2D and 3D cultures. COL1, COL4 and FN (2D cultures) and COL6, FN and αSMA (3D cultures), and FGF-2 also caused significantly different effects, namely, a significant down-regulation (2D cultures) and a significant up-regulation (3D cultures), respectively, on the TGF-β2 untreated or treated cells. While in contrast, only a significant up-regulation of the immunostaining of COL6 by FGF-2 in the 3D HconF spheroids was observed (Fig. 5), although those for the 2D cultures were similar to the results of qPCR analysis (Supplemental Fig. 2). In terms of the diversity between qPCR and the immunolabeling of the 3D spheroids, this was also reported in our previous study using HTM cells^29,35^ as well as other sources of cells^28,34,36^. As a possible explanation for this, we speculate that immunolabeling may adequately reflect the expression of target molecules that are located on the surface of the 3D spheroids while, in contrast, the total expression detected by qPCR analysis. Similar to ECM proteins, differences between 2 and 3D cultured HconF cells were also observed for the mRNA expression of *TIMP1-4* (Fig. 6A), *MMP2, 9* and *14* (Fig. 6B), ER-stress related factors including the glucose regulator protein (GRP)78, GRP94, the X-box binding protein-1 (XBP1), spliced XBP1 (sXBP1) and CCAAT/enhancer-binding protein homologous protein (CHOP) (Fig. 7). Nevertheless, a rational correlation between these TGF-β2 and/or FGF-2 effects on the expression of ECM and the expression ECM modulators, TIMPs (3D; significant up-regulations of TIMP 1 and 2) and MMPs (2D; TGF-β2 induced a substantial up-regulation of MMP2, which was suppressed by FGF2, 3D; a significant up-regulation of MMP9 and the down-regulation of MMP14 in the presence or absence of TGF-β2) were not observed. However, in the case of the 5 ER-stress related factor, the expression of XBP (2D cultures) and CHOP (3D cultures) were significantly down-regulated and up-regulated by FGF-2, and slight additive effects by TGF-β2 and FGF-2 were observed for Grp94, XBP and CHOP (2D cultures) and all 5 genes, except for XBP (3D cultures). These collective findings indicate that some of the expressions of the ECMs and their regulatory factors, TIMPs and MMP, as well as ER-stress related factors were altered by FGF-2, and these FGF-2 induced effects were also modulated by the simultaneous presence of TGF-β2, suggesting that both factors may induce some additive effects, as observed in several of the above biological analyses in the 2D and 3D cultured HconF cells.

## Discussion

Our recent studies using 2D and 3D cell culture systems demonstrated that 3D spheroids have significantly different biological properties in their function and in the expression of several cellular components of molecules such as ECM proteins, their modulators including TIMPs, MMPs and ER stress related factors as compared with conventional 2D planer cultures of adipocytes^34,37^, corneal scleral fibroblasts^38^, human trabecular meshwork cell^35,39,40^, human corneal fibroblasts^41^, in addition to HconF cells^26^. Among these, we reported that adipogenic differentiation was much more efficient in the case of 3D 3T3-L1 spheroids as compared to 2D cultured 3T3-L1 preadipocyte^34,42–45^. In addition, most recently, to gain insights into the possible mechanisms responsible for such biological differences between 2 and 3D cell cultures of 3T3-L1 cells, we carried out a RNA sequence analysis^46^ and a bioinformatic analysis using Gene Ontology (GO) enrichment analysis and, in an ingenuity pathway analysis (IPA), we identified 6 genes, TGFβ1, STAT3, IL6, AGT, FOS and MYC, as upstream regulators^37^. Therefore, these collective observations suggest that 3D spheroid cultures may be powerful and useful in terms of elucidating unidentified biological aspects of cells that cannot be determined when conventional 2D cultures are used. In fact, our recent investigation suggested that 2D cell cultures and 3D spheroid cultures of TGF-β2 treated HconF cells may replicate different pathogenic conditions, that is, planer and subepithelial fibrogenesis^26^, based upon biophysical analyses as well as analyses of the expression of ECM proteins, their modulators including TIMPs, MMPs and ER stress related factors.

In terms of the effects of FGF-2 on the TGF-β-induced transdifferentiation of HconF cells, Matsumura et al. recently reported that FGF-2 attenuated the TGF-β-induced expression of α-SMA in HconF cells^25^. In addition, since such FGF-2 induced effects were also observed on a soft substratum culture well, on which the TGF-β-induced expression of α-SMA was greatly affected, FGF-2 induced attenuation independently occurred for TGF-β as well as in the case of mechanical stress. In the present study, to investigate this issue further, we employed our recently developed 2D and 3D cultures of TGF-β2 treated HconF cells as representative in vivo conjunctival models for fibrogenetic changes on the plane or in the spatial space, respectively, and obtained the following different results; FGF-2 and TGF-β2 induced (1) significant increase in the planar proliferation of the 2D HconF cell monolayers, as evidenced by TEER values and FITC dextran permeability, (2) a shift in the metabolic phenotype of the 2D cultured HconF cells toward a more energetic state, (3) the formation of enlarged and stiffer 3D HconF spheroids. Therefore, these collective findings suggest that FGF-2 significantly modulated the TGF-β2-induced increase in fibrogenetic changes in both 2D and 3D cultured HconF cells. In fact, this conclusion is supported by data reported in a previous study by Kay et al.^24^. In that study, they concluded that FGF-2 may be the direct stimulator of TGF-β-mediated cell proliferation in rabbit conjunctival fibroblasts.

Although we currently have no explanation for these conflicting results, differences in the methodology used for the evaluation of the fibrogenetic changes may be a factor. Matsumura et al. performed a planar culture with a 48-h incubation time, which is a different system from our 6-day incubation time. In addition, they mainly used western blot analysis and immunohistochemistry in their characterization studies, but, in our study, we performed TEER and FITC-dextran permeability measurements (2D cultures), real-time cellular metabolic analyses (2D cultures), qPCR and immunocytechemistry (2D and 3D) and a unique analysis of the physical properties, size and stiffness of the 3D spheroids despite the fact that the source of the cells was identical, as were the drug concentrations. It is also noteworthy that, in terms of effects related to mechanical, they indirectly evaluated this using silicone plates with different degrees of stiffness and focused on YAP/TAZ, key transducers of mechanical stress, while our group directly measured the stiffness of the 3D HconF spheroids. In fact, our unique and straightforward technique in which a micro-squeezer is used to directly compress a single living 3D spheroid has been shown to be quite reproducible and reliable. In fact, in addition to the application of this method to HconF cells, this method has also been confirmed to be useful for estimating the stiffness of cells from other sources, including human orbital fibroblast^27,28,47^, mouse preadipocytes^34,42,43,45^, human trabecular meshwork cells^29,35,39,48^, as well as others. Furthermore, in our current study, the mRNA expression of all ECMs including *COL1, 4* and *6, α-SMA* and *FN* were significantly increased upon administering 10 nM FGF-2. While, in contrast, western blot analysis by Matsumura et al. indicated that FGF-2 caused almost no effects on the expression of COL1, FN and α-SMA, and on the suppressive effects toward the TGF-β2-induced increase in α-SMA expression^25^.

Several clinical studies have shown that FGF-2 functions to exert anti-fibrotic effects under conditions as diverse as burns, chronic wounds, oral ulcers, vascular ulcers, diabetic ulcers, pressure ulcers, and surgical incisions^49–52^. In contrast, the effects of FGF-2 within conjunctival tissues remains quite controversial, as described above. However, the current study has some limitations, as follows. Although only a few related studies regarding this issue have appeared so far, several diverse effects of FGF-2 toward TGF-β2 induced myofibrogenic phenotypes were reported among several different tissues (suppression; orbital fibroblast^53^, Valvular interstitial cells^54^, stimulation; lens epithelial cells^55^). In addition, the underlying mechanisms for causing some additive effect by FGF-2 and TGF-β2 as observed in the biophysical analyses of the above 2D and 3D HconF cells remain to be elucidated despite our efforts to closely examine the several candidate molecules. Therefore, to better understand the results related to the role of FGF-2 on TGF-β2-induced fibrogenetic changes of 2D and 3D cultured HconF cells, further investigations, including an RNA-Seq experiment will be needed to elucidate possible mechanisms linked with several other complex signaling networks in order to confirm our present observations, since quite recently, in an RNA-Seq study, the FGF-2-responsive transcriptional profile was comprehensively analyzed and the findings revealed potential candidates for mechanisms in terms of FGF-2-mediated wound healing using skin fibroblasts^56^.

## Supplementary Information

Supplementary Information 1.

Supplementary Information 2.

## Data Availability

The datasets used and/or analyzed during the current study avairable from the corresponding author on reasonable request.
