# Supplementary material for: FGF-2 enhances fibrogenetic changes in TGF-β2 treated human conjunctival fibroblasts
Source: Sci Rep. 2022 Sep 26;12:16006. doi: 10.1038/s41598-022-20036-7 (PMC9512844; doi:10.1038/s41598-022-20036-7)
Supplement: Supplementary file 1 — Supplementary Information 1. [file 41598_2022_20036_MOESM1_ESM.pdf]

FGF-2 enhances fibrogenetic changes in TGF-β2 treated human conjunctival fibroblasts

Yuri Tsugeno[1], Masato Furuhashi[2], Tatsuya Sato[2,3], Megumi Watanabe[1], Araya Umetsu[1], Soma Suzuki[1], Yosuke Ida[1], Fumihito Hikage[1], Hiroshi Ohguro[1].

Departments of Ophthalmology[1], Cardiovascular, Renal and Metabolic Medicine[2] and Cellular Physiology and Signal Transduction[3], Sapporo Medical University School of Medicine

Short title: Study of TGF-β2 treated 2D and 3D cultured HconF cells

Key words: TGF-β2, human conjunctival fibroblast, 3D culture, FGF-2.

All correspondence should be addressed to Hiroshi Ohguro

Tel# 81-11-611-2111, Fax# 81-11-613-6575, e-mail: ooguro@sapmed.ac.jp

Three authors (Y.T., M.F. and T.S.) contributed equally to this manuscript.

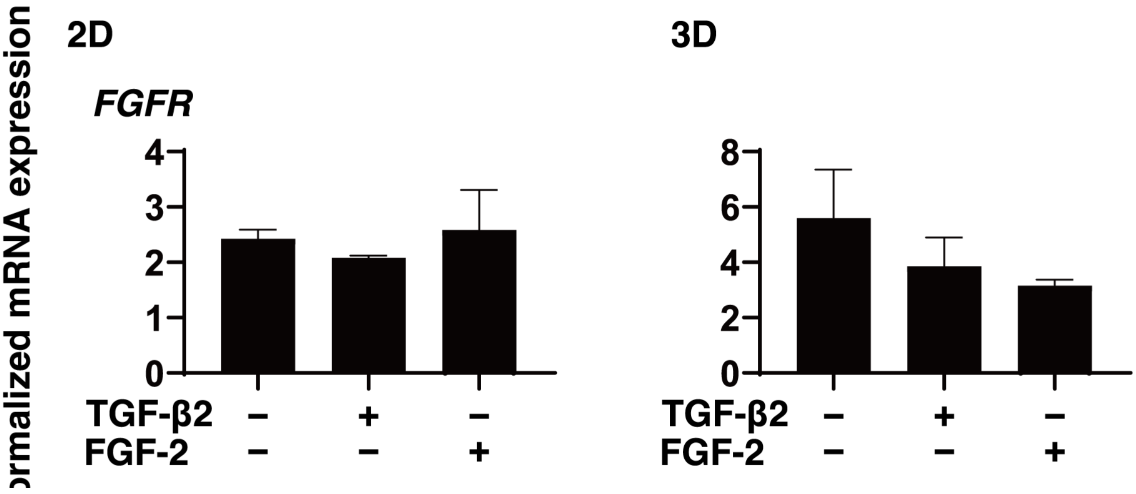

Supplemental Figure 1. Effects of FGF-2 on mRNA expression of FGFR of 2D and 3D cultured HconF cells in the absence or presence of TGF-β2.

2D and 3D HconF cells were treated with a 5 ng/ml solution of TGF-β2 in the absence or presence of 10 ng/ml FGF-2 with an untreated sample as the control, and at Day 6 each sample was subjected to qPCR analysis and the expression of mRNA in *FGFR* were estimated. All experiments were performed in duplicate using 3 different confluent 6-well dishes (2D) or 15 freshly prepared 3D HconF spheroids (3D) in each experimental condition. Data are presented as the arithmetic mean ± the standard error of the mean (SEM).

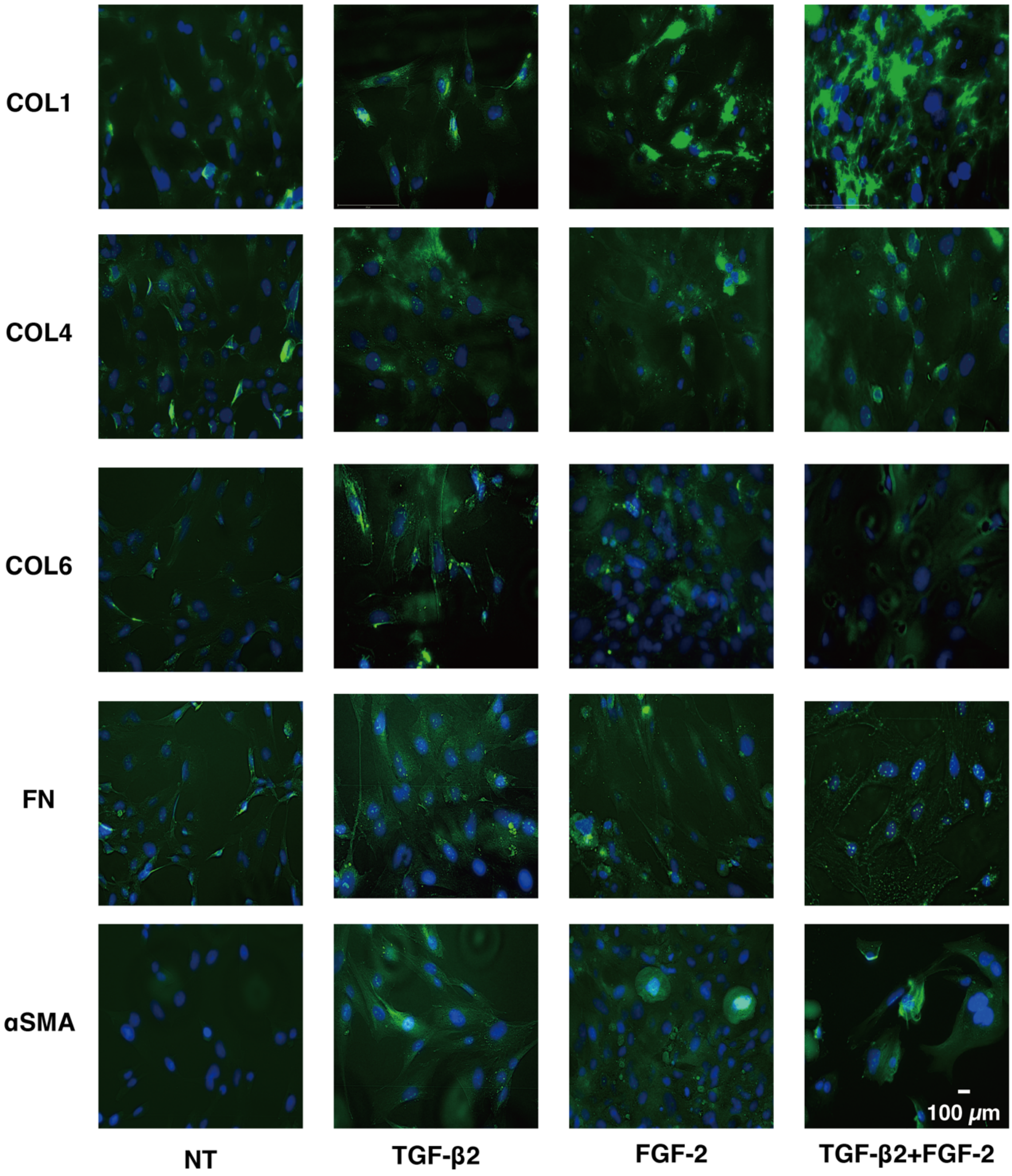

Supplemental Figure 2 Immunolabeling of ECMs of the 2D HconF sphenoids.

2D cultured HconF cells were treated with a 5 ng/ml solution of TGF-β2 in the absence or presence of 10 ng/ml FGF-2 with an untreated sample as the control, and at Day 6, each sample was subjected to immunostaining for *COL 1*, *COL 4*, *COL 6*, *FN* and *a-SMA*. All experiments were performed in duplicate using fresh preparations (n=5). Representative images are shown.
